# Longitudinal detection of somatic mutations in the saliva of head and neck squamous cell carcinoma–affected patients: a pilot study

**DOI:** 10.3389/fonc.2024.1480302

**Published:** 2024-11-01

**Authors:** Chiara Dal Secco, Alessandro Tel, Lorenzo Allegri, Federica Baldan, Francesco Curcio, Salvatore Sembronio, Flavio Faletra, Massimo Robiony, Giuseppe Damante, Catia Mio

**Affiliations:** ^1^ Department of Medicine (DMED), University of Udine, Udine, Italy; ^2^ Clinic of Maxillofacial Surgery, Head-Neck and NeuroScience Department, University Hospital of Udine, Udine, Italy; ^3^ Department of Laboratory Medicine, Institute of Clinical Pathology, University Hospital of Udine, Udine, Italy; ^4^ Department of Laboratory Medicine, Institute of Medical Genetics, University Hospital of Udine, Udine, Italy

**Keywords:** head and neck squamous cell carcinoma, saliva, next generation sequencing, liquid biopsy, relapse

## Abstract

**Introduction:**

Liquid biopsy is gaining momentum for diagnosis and surveillance of cancer patients. Indeed, head and neck squamous cell carcinoma (HNSCC) is burdened with poor prognosis and high recurrence rates after treatment. It is therefore crucial to be able to detect minimal residual disease early after radical treatment or relapse, so surgery can be performed when the disease is still resectable. In this scenario, aim of this study is to create a liquid biopsy-based pipeline able to detect somatic tumor mutations in a cohort of HNSCC-affected patients undergoing follow-up after surgical intervention.

**Methods:**

Our cohort included 17 patients diagnosed with HNSCC over 4 years. The first saliva sample was collected before surgery while the rest were collected during the subsequent visits, according to the follow-up schedule. Salivary DNA (sDNA) was extracted, and a 52-gene next generation sequencing (NGS)-based panel was used for somatic variants detection.

**Results:**

41.2% of samples collected before surgery bore a deleterious variant (n=7/17). Overall, 29.2% of samples harbored at least a pathogenic variant (n=21/72). The most frequently mutated genes were TP53 (80%), FBXW7 (8%), PDGFRA (4%) and PTEN (4%). Finally, three patients experienced a loco-regional relapse by clinical evaluations, anticipated in 67% of cases by the molecular one (n=2/3).

**Discussion:**

Our data indicate that sDNA could aid in the monitoring of patients’ follow-up as low-frequency somatic mutations could be assessed from the saliva of HNSCC patients. Prospectively, these results suggest that salivary-based liquid biopsy might pave the way for personalized molecular therapies based on mutational data.

## 1 Introduction

Head and neck squamous cell carcinoma (HNSCC) is an umbrella term that groups malignancies arising from the oral cavity, pharynx, hypopharynx, larynx, nasal cavity, and salivary glands. It is the seventh most common cancer diagnosis worldwide, with an incidence of 20 per 100,000 adults (1). The gold standard for early detection of HNSCC still remains clinical examination (i.e., inspection and palpation-related maneuvers). However, the mainstay of diagnosis is represented by invasive surgical biopsy.

To date, tobacco smoking and alcohol drinking behaviors are major risk factors. Additionally, human papillomavirus (HPV) infection is an important risk factor predominantly for oropharyngeal cancer (2). Remarkably, HPV-positive carcinomas are a well-characterized entity (3, 4), primarily affecting non-smoking young or middle-aged males. They generally have a more favorable prognosis than HPV-unrelated tumors (5, 6).

HNSCCs are certainly one of the worst stratified carcinomas: the overall survival is 50% at five years, but varies enormously (10%-80%) in relation to tumor size and biological characteristics, with hypopharynx cancers experiencing the worst outcomes (7). It would be essential to differentiate tumors with a more aggressive biological behavior at the time of diagnosis, implementing the actual TNM staging system used in clinical practice. Several prognostic models have been proposed, but none of these has entered the clinics. These models, often based on small non-selected cohorts of patients, rely on solely clinical features, ignoring the tumor-related molecular profile that is paramount to deliver the proper treatment to each patient.

In the last 15 years, oncogenomics has been boosted with rapid advancements in next-generation sequencing (NGS) technologies. Both genetic and epigenetic information could be assessed, and this might provide important knowledge about tumor biology. Although molecular heterogeneity is a hallmark of HNSCC, some genes were found to be frequently altered such as TP53 (67.5% of analyzed cases), CDKN2A (43.7%), PIK3CA (32.5%), followed by less frequently altered genes such as EGFR, PTEN and HRAS (8).

Liquid biopsy is shaping a novel approach for the management of cancer patients. It is well-known that circulating tumor DNA (ctDNA) is released from primary tumors and metastatic sites into body fluids as peripheral blood, saliva, urine, feces and cerebrospinal fluid (9). Indeed, ctDNA is a combination of highly fragmented nucleic acids with a half-life that ranges from 16 minutes to 2.5 hours, thus making it a good tool for dynamic monitoring of the disease (10). Over the past decade, several studies have demonstrated the potential applications of ctDNA analysis for shaping patients’ care in non-small cell lung cancer, colorectal and breast cancer (11–13).

The identification of biomarkers from biological fluids (i.e., plasma and saliva) has the potential of simplifying diagnosis, and providing an overall estimation of the disease burden. In particular, the use of saliva for both early cancer diagnosis and relapse detection represents a promising approach in the search for new clinical markers, given its noninvasive and easy-to-collect nature (14, 15). It represents a readily available biological matrix that may provide useful information to anticipate detection, diagnosis, profiling, prognosis and follow-up of the disease. Although ctDNA is often present at frequencies <1% of all DNA in saliva samples, remarkable advances in NGS technologies (such as incorporation of molecular tagging and advancements in algorithms for background noise suppression) has made it a highly sensitive and specific platform for low variant allele frequency (VAF) mosaic variants detection (15). In light of such considerations, liquid biopsy has the potential to become the new mainstay in the diagnosis of oral cancer.

Given the premises, aim of this study is to create an analytical pipeline able to detect somatic tumor mutations evaluating a cohort of 17 patients affected by HNSCC undergoing follow-up after surgical intervention.

## 2 Materials and methods

### 2.1 Patient’s samples

This study uses clinical information and biological samples from 17 individuals referred to the Clinic of Maxillofacial Surgery within the Head-Neck and NeuroScience Department at the University Hospital of Udine (Italy) over 4 years (October 2019-October 2023). The study was approved by the Institutional Review Board of the University of Udine (RIF. Prot IRB: 177/2024) and performed in accordance with the Declaration of Helsinki. Individual written informed consent was obtained from all participating patients at enrolment.

Participants were limited to those diagnosed with non-metastatic disease treated with curative intent. Curative intent includes any combination of surgery, radiotherapy and chemotherapy. Main inclusion criteria were: i) ≥ 18 years, ii) histological confirmation of squamous cell of the head and neck (stages I to IV by AJCC 8th edition), iii) deemed resectable and scheduled for surgery. Main exclusion criteria were: i) presence of distant metastasis, ii) surgical procedure in the last three days or iii) previous history of another active neoplasm in the last 5 years.

5 mL of saliva samples were collected in DNA/RNA Shield SafeCollect™ Saliva Collection Kit (R1211, Zymo Research), following manufacturer instructions. The first time point, here called T0, was collected before surgery while subsequent ones (i.e. T1, T2, T3 and so on) were collected during the following visits according to the follow-up schedule.

### 2.2 Salivary DNA extraction

5 mL saliva samples were collected in the DNA/RNA Shield SafeCollect™ Saliva Collection Kit (R1211, Zymo Research), following manufacturer instructions, prior surgery and at clinical follow-up. Patients were asked to avoid eating, drinking, or performing oral hygiene for at least one hour prior to collection (16). First, cellular debris were removed from samples by centrifuging at 16000g for 10 minutes. Cell-free saliva samples were stored at -20°C until usage.

sDNA was isolated using the Sera-Xtracta™ Cell-Free DNA Kit (29437807, Cytiva) following manufacturer’s instructions. Briefly, Proteinase K (20 mg/mL) and 20% SDS were added to 1 ml of saliva and incubated at 60°C for 20 min. Then, 2.1 mL of binding mix containing magnetic beads were added to each sample, mixed thoroughly by pulse vortexing, and incubated for 10 min at room temperature in shaking (1000 rpm). Samples were then placed into a magnetic rack and, after washing, sDNA was eluted from the beads adding 50 µL of elution buffer and incubating for 3 min at room temperature in shaking (1400 rpm). Finally, samples were placed into the magnetic rack in order to collect the supernatant containing the isolated sDNA. Samples were quantified using the Qubit dsDNA HS Assay kit (Life Technologies) on a Qubit 4.0 instrument (Life Technologies).

### 2.3 Library preparation and next generation sequencing

A total of 79 samples out of 17 patients were processed. 30 or 50 ng of sDNA were used for sequencing. The Oncomine™ Pan Cancer Cell Free Assay (A37664, ThermoFisher) was used for library preparation, following manufacturer’s protocol. The 52-gene panel is designed to target single nucleotide variants (SNVs), copy number variants (CNVs) and gene fusions with a limit of detection (LOD) down to 0.065%, >80% sensitivity and >98% specificity.

Libraries were manually prepared and all reactions were performed in a VeritiPro™ 96-Well Thermal Cycler (Applied Biosystems). Libraries were pooled in 4-plex and loaded into the Ion Chef™ Instrument (Thermo Fisher Scientific) for template enrichment and chip loading. Sequencing was performed on the Ion S5 GeneStudio Sequencer using the Ion 540™ chip (Thermo Fisher Scientific).

### 2.4 Data analysis

Sequencing data were processed using the Torrent Suite 5.20.2.0 software pipeline to perform raw data analysis, base calling, removal of low-quality reads, and alignment to the human genome (GRCh37/hg19). Variant calling was performed with Ion Reporter 5.16. Only samples with a molecular coverage ≥ 2500X, with a variant allele frequency (VAF) ≥ 0.1% and with a p-value ≤ 0.05 were considered suitable for the analysis. Variants with frequency <1% in population-based databases (i.e., gnomAD), exonic missense, splicing, stop-gain, stop-loss, and frameshift insertion and deletion variants were retained for further evaluation. The following public databases were used for the interpretation of the variants: HGMD Professional (https://my.qiagendigitalinsights.com/bbp), ClinVar (https://www.ncbi.nlm.nih.gov/clinvar/) and Franklin by genoox (https://franklin.genoox.com/clinical-db/home).

Variants were classified according to the American College of Medical Genetics and Genomics/Association for Molecular Pathology (ACMG/AMP) guidelines (17, 18) and the joint recommendations of Clinical Genome Resource (ClinGen), Cancer Genomics Consortium (CGC) and Variant Interpretation for Cancer Consortium (VICC) (19).

### 2.5 Statistical analysis

Data are shown as mean ± standard error of mean (SEM). Statistical significance was assessed by one-way ANOVA test with Bonferroni correction performed with Prism v6 (GraphPAD Software for Science).

## 3 Results

### 3.1 Clinical characteristics of HNSCC patients

A cohort of 17 patients was enrolled in this prospective, longitudinal pilot study. All patients were diagnosed with squamocellular carcinoma of the head and neck, further classified in the following sub-regions: gingival mucosa of the upper maxilla, or maxillary infiltration from cancer originating in paranasal sinuses (4 patients), gingival mucosa of the mandible and the retromolar trigone (4 patients), tongue (6 patients), floor of the mouth (1 patient), and inner mucosa of the cheek (2 patients). Mean age was 65, 5 years (SD: 11,3 years), with 11 males and 6 females. At enrolment, risk factors for HNSCC were assessed, highlighting 11.76% of HPV-positive samples (n=2/17), 58.82% with a positive smoking history (n=10/17) and 17.65% with a documented alcohol history (n=3/17) (**Supplementary Table S1**).

During the time interval considered by this study, one patient died for progression of disease in metastatic stage IV. All patients underwent radical surgical excision with curative intent; 6 patients underwent combined radiation therapy and chemotherapy, 4 patients underwent radiation therapy alone, 2 patients were candidate to third line immunotherapy owing to disease progression, whereas 5 patients did not undergo any adjuvant therapy in relation to the histological characteristics of the excised specimen. All patients were subjected to initial 2-year monthly clinical follow-up program.

### 3.2 Detection of DNA variants in saliva samples

**Figure 1:**
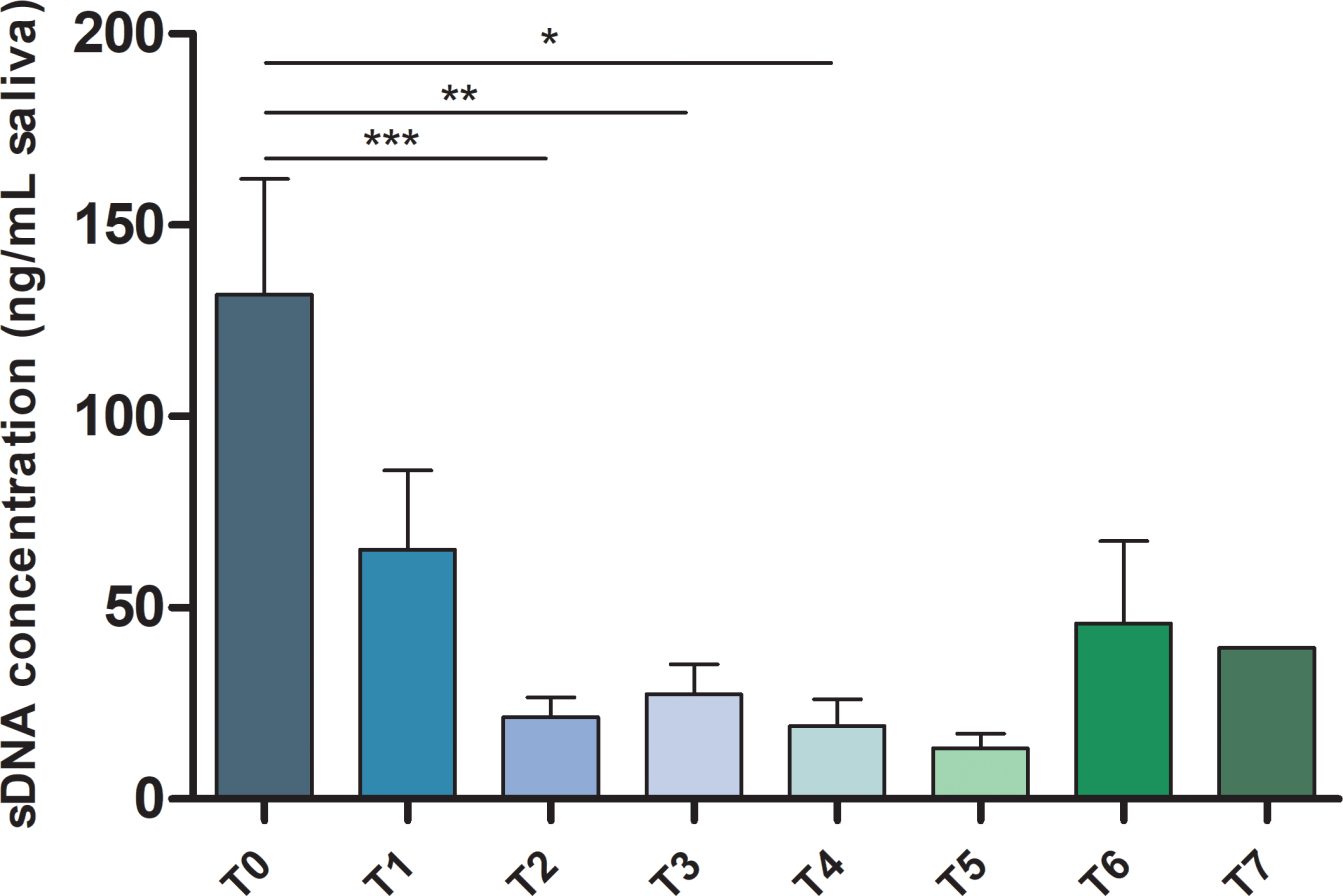
sDNA concentration in HNSCC patients’ samples. Bar chart showing the mean ± SEM of sDNA concentration for each analyzed time point from T0 to T7. *p-value ≤ 0.05; **p-value ≤ 0.01; ***p-value ≤ 0.001.

A total of 79 samples were collected from 17 patients at different time points, i.e., prior surgery and at clinical follow-up. The mean number of time-points collected for each patient was 4.65 [min 3 - max 8]. As expected, a significant higher sDNA concentration was detected in T0 time-points compared to subsequent ones (**Figure 1**). It should be mentioned that in 3/17 T1 samples the sDNA concentration was higher than in T0 (**Supplementary Figure S1**). This phenomenon is probably due to the fact that surgical demolition and reconstruction was performed shortly before saliva collection.

Subsequently, targeted NGS was performed to depict the molecular landscape characterizing our cohort. Mean molecular coverage was 5078X ± 1669X. Six samples failed QC test and were omitted from analysis. Variants below the limit of detection (LoD) were labelled as artefacts. After quality-based filtering, 29.2% of samples harbored at least one deleterious somatic variant (n=21/72). Comprehensively, 52.9% of patients turned out to bear at least one pathogenic somatic variant in any of the tested samples (n=9/17). Furthermore, 41.2% of samples collected before surgery (T0) bore a deleterious variant (n=7/17).

Analyzing SNVs, the most frequently mutated genes were *TP53* (80%), *FBXW7* (8%), *PDGFRA* (8%) and *PTEN* (4%). The most prominent mutation types were missense mutations (76%), the rest were frameshift ones (24%) (**Table 1**).

**Table 1:** SNVs detected in our cohort assessed by next generation sequencing.

Gene	Transcript	Nucleotide change	Amino acid change	ACMG classification	Oncogenic classification	n° samples (n° patients)
*TP53*	NM_000546.6	*c.294_298del*	*p.Ser99GlufsTer48*	*Pathogenic*	Likely Oncogenic	*1 (1)*
*TP53*	NM_000546.6	*c.568C>A*	*p.Pro190Thr*	*Likely Pathogenic*	Likely Oncogenic	*1 (1)*
*TP53*	NM_000546.6	*c.635_636del*	*p.Phe212SerfsTer3*	*Pathogenic*	Oncogenic	*1 (1)*
*TP53*	NM_000546.6	c.659A>G	p.Tyr220Cys	Pathogenic	Oncogenic	8 (3)
*TP53*	NM_000546.6	*c.712T>G*	*p.Cys238Gly*	*Pathogenic*	Oncogenic	*1 (1)*
*TP53*	NM_000546.6	c.713G>A	p.Cys238Tyr	Pathogenic	Oncogenic	1 (1)
*TP53*	NM_000546.6	*c.733G>A*	*p.Gly245Ser*	*Pathogenic*	Oncogenic	*1 (1)*
*TP53*	NM_000546.6	*c.743G>T*	*p.Arg248Leu*	*Pathogenic*	Oncogenic	*2 (1)*
*TP53*	NM_000546.6	c.749C>T	p.Pro250Leu	Pathogenic	Likely Oncogenic	1 (1)
*TP53*	NM_000546.6	*c.759delC*	*p.Ile254SerfsTer91*	*Pathogenic*	Oncogenic	*2 (1)*
*TP53*	NM_000546.6	*c.824G>T*	*p.Cys275Phe*	*Pathogenic*	Oncogenic	*1 (1)*
*FBXW7*	NM_033632.3	*c.1513C>T*	*p.Arg505Cys*	*Pathogenic*	Likely Oncogenic	*2 (2)*
*PDGFRA*	NM_006206.6	c.1396_1397insT	p.Ala466ValfsTer39	VUS	Uncertain	2 (1)
*PTEN*	NM_000314.8	c.274G>A	p.Asp92Asn	Likely Pathogenic	Moderate Oncogenic Support	1 (1)

VUS, variant of unknown significance.

**Figure 2:**
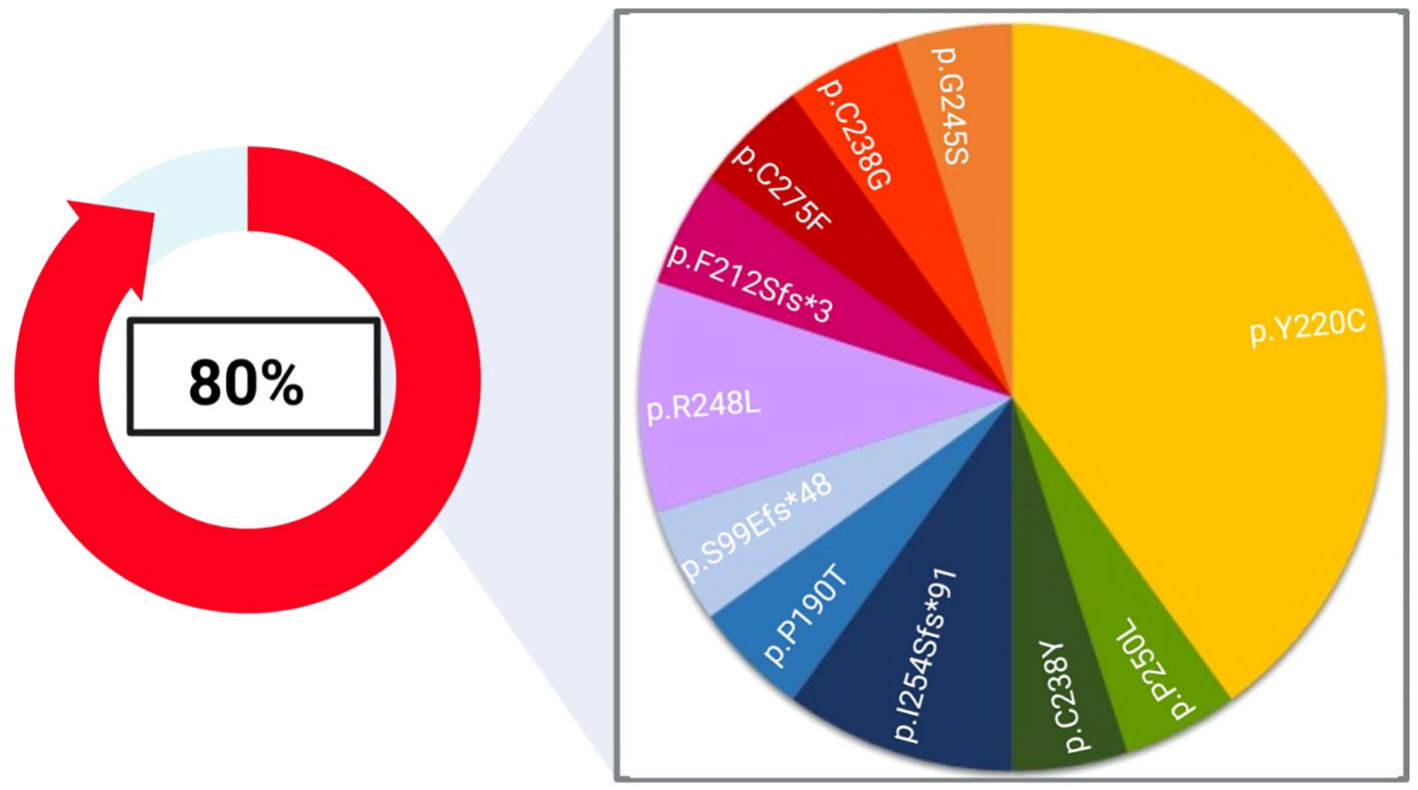
Landscape of deleterious TP53 alterations found in our cohort. 80% of the single nucleotide variants (SNVs) highlighted in this study affected the *TP53* coding sequence and were assessed in 8 out of 17 patients. The pie chart represents the frequency of each variant detected in this study. Created with Biorender.com.

As summarized in **Figure 2**, the most recurrent deleterious TP53 variant involved the codon 220 (p.Y220C), detected in 40% of TP53 positive samples. Indeed, this variant is strongly associated with squamous cell cancers of the head and neck (20).

All the mutations detected were classified as deleterious except for the PDGFRA p.Ala466ValfsTer39 variants that have been classified as VUS/Uncertain, following the most recent variant classifications (17–19).

Besides SNVs, three T0 samples displayed deleterious CNV, i.e., amplification of either *FGFR3* (n=2/17) and *CCDN1* (n=1/17).

**Supplementary Table S2** summarizes genetic findings assessed in our cohort.

### 3.3 sDNA analysis anticipates clinical relapse

Since tumour relapse is one of the hallmark of HNSCC, in a subsequent analytical step the ability to anticipate clinical recurrence was investigated. Summarizing clinical and molecular data, 18% of the patients received a diagnosis of loco-regional relapse by clinical evaluations and in 67% of cases clinical recurrence was anticipated by the molecular one (n=2/3).

**Figure 3:**
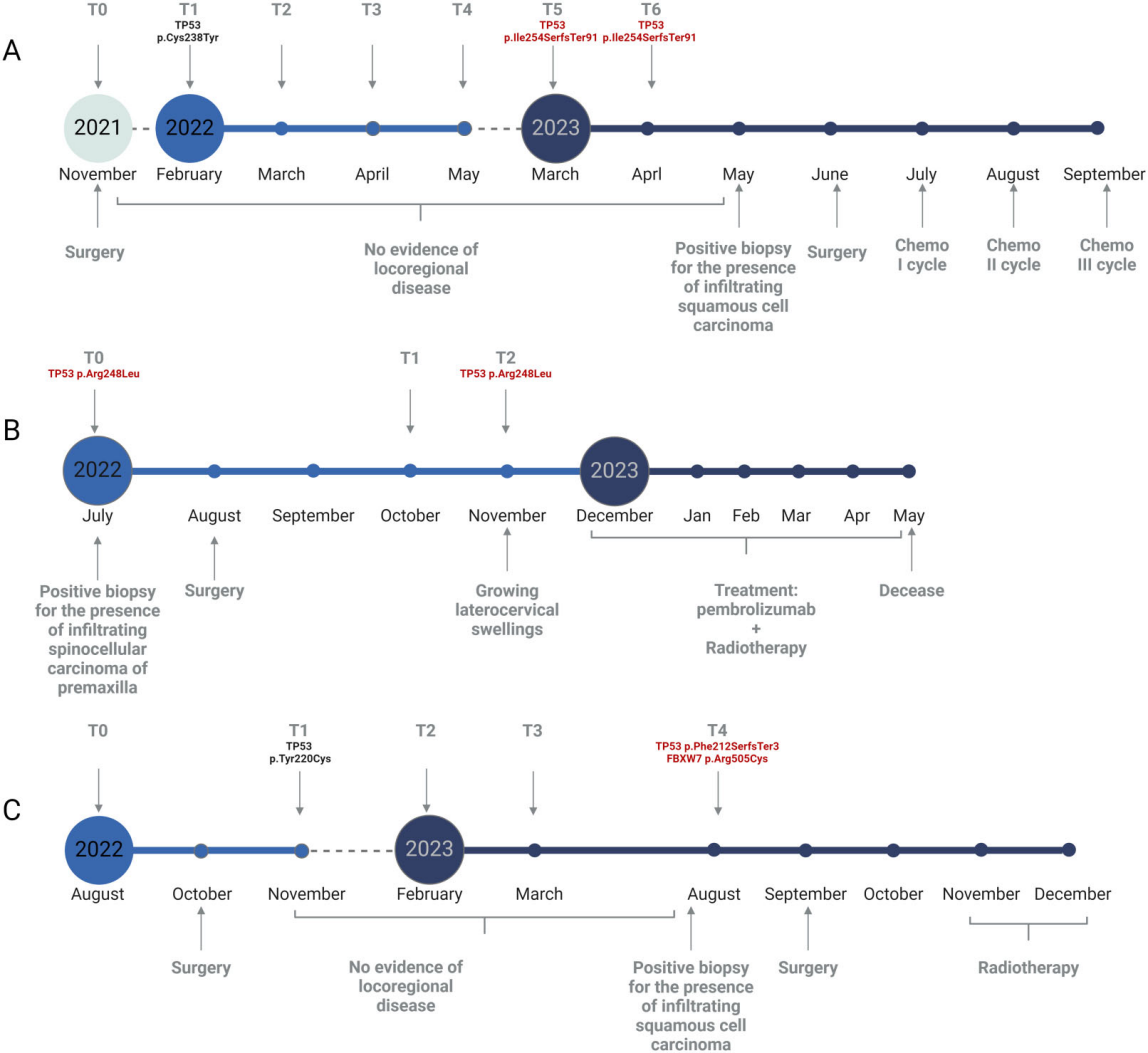
Graphical representation of relapsed patients’ follow-up. **(A)** Timeline for patient HNSCC_02. **(B)** Timeline for patient HNSCC_15. **(C)** Timeline for patient HNSCC_05. Time points of saliva collection are depicted as grey arrows in the upper section of each panel. Clinical data are reported in the lower section of each panel. Somatic variants detected with our NGS approach are reported in red. Created with Biorender.com.

Patient HNSCC_02 did not harbor any recurrent deleterious variant from T0 to T4. Yet, at T5 and T6 a pathogenic TP53 p.Ile254SerfsTer91 variant was detected, with an increasing VAF of 0.23% and 0.45%, respectively. A clinical diagnosis of tumour relapse was made 4 months after the first molecular occurrence (**Figure 3A**).

Patient HNSCC_15 presented a pathogenic TP53 p.Arg248Leu mutation at T0 (VAF= 1.50%), which was not assessed in T1 but recurred at T2 (VAF= 0.14%), anticipating the clinical diagnosis of relapse. Unfortunately, the recurrence was highly aggressive and led to the patient’s exitus before any surgical intervention could take place (**Figure 3B**).

Finally, patient HNSCC_05 did not bear any recurrent deleterious variant from T0 to T3. Thereafter, two novel mutations occurred at T4: a pathogenic TP53 p.Phe212SerfsTer3 (VAF= 0.21%) and a likely pathogenic FBXW7 p.Arg505Cys (VAF= 0.15%). Clinical relapse was diagnosed soon before the molecular one (**Figure 3C**).

## 4 Discussion

Head and neck squamous cell carcinoma (HNSCC) is a relatively common malignancy with poor prognosis and a high mortality rate (21). Biopsy is the first choice for diagnosis of malignant lesions. It is an invasive procedure that has its own drawbacks and is practically not possible for terminally ill patients (22). Despite technological improvements and advances achieved in the therapeutic approach of these malignancies, the incidence of tumour relapse remains steadily at 15-50% (23).

Circulating DNA from liquid biopsy is gaining momentum in cancer diagnostics as a rapid marker of recurrence in various cancer types (9). In particular, saliva became a matrix of high interest in the field of head and neck cancers since its close connection with the districts affected by the disease. In a scenario where the overall survival is less than 50% at 5 years (24), the analysis of saliva-related biomarkers is achieving considerable impact in the context of molecular relapse (25).

Saliva is a complex fluid containing proteins, metabolites, DNA, RNA and microbiota that can be easily used as biomarkers (26). The utility of saliva as a potential diagnostic fluid offers a great amount of benefits beyond its non-invasiveness, such as the ease of accessibility and convenience for repeated collections (27).

In this study, we aimed at creating an analytical pipeline able to detect somatic tumor mutations in saliva samples of a cohort of 17 patients affected by HNSCC, with the ultimate goal of monitoring minimal residual disease and foreseeing tumor relapse. For this purpose, a 52-gene panel was used and saliva samples were collected after surgery and at scheduled follow-up. The choice to monitor patients with this approach even after the driver mutation is identified at T0 lies in the fact that it can cover the evolution of the tumor mass and detect dynamic changes related to intra-tumor heterogeneity.

In fact, we successfully detected somatic mutations in patients’ saliva with great resolution, not only in T0, where the tumor burden is at its highest, but also during subsequent follow-up. The great majority of patients harboring a deleterious variant before surgery did not show any molecular or clinical signs of disease recurrence during the 4-years longitudinal study. Notwithstanding, 18% of patients experienced a clinically evaluated loco-regional relapse, in most cases anticipated by the detection of a deleterious variant by sequencing (n=2/3).

In our cohort, TP53 was the most prevalently altered gene (47%, n=8/17), corroborating data already published in diverse studies (20, 22, 25, 28, 29). Indeed, TP53 is the most frequently mutated tumour suppressor gene among HPV-negative HNSCC cases, with a positivity rate ranging from 75% to 85% (28). Indeed, the mutational profile of TP53 has been recognized as an independent prognostic factor in HNSCC (30), which can stratify patients into low- and high-risk survival rates, the latter being the only group at a higher risk of treatment failure (31). Overall, 80% of samples bore at least a SNV in TP53, with the p.Tyr220Cys being the most frequent TP53 mutation identified in our cohort. This variant is a well-established early marker of cancer progression (20).

Genetic factors affecting the etiology of HNSCC include not only SNVs, but also copy number variations (CNVs) (32). Sequencing data show that three patients bore a pathogenic amplification of either *FGFR3* or *CCDN1*. Indeed, amplification of *FGFR3* and *CCDN1* have been independently reported to be associated to oral carcinoma and both represent a biomarker of poor prognosis (33–36).

We, then, explored the ability of our NGS-based liquid biopsy approach to detect the clinical recurrence in cancer patients. Throughout this study, three patients experienced a relapse.

The clinical history of HNSCC_02, a 56 years old male, starts in June 2020 when he was diagnosed with infiltrating squamocellular carcinoma of the tongue, which was immediately treated with surgical resection. Unfortunately, the patient undergoes clinical relapse within 6 months after the first diagnosis. The patient was then enrolled in our study and the first saliva sample was collected before the surgical removal of the first relapse. NGS analysis of sDNA showed no evidence of molecular recurrence as no pathogenic variant was found for about 16 months. In March 2023, corresponding to T5, a pathogenic TP53 variant was assessed with a VAF of 0.2%, confirmed with a higher frequency one month later (T6, 0.45%). Surprisingly, the molecular recurrence was not matched by an increase in the amount of sDNA. In May 2023, about three months after the first occurrence of a pathogenic mutation, a clinical relapse was suspected and then confirmed with a tissue biopsy (**Figure 3A**).

An even more striking example of this trend is found analyzing HNSCC_15, an 82 years old female. The patient was sent to the Clinic of Maxillofacial Surgery as she presented gingival algias. The clinician immediately made a diagnosis of premaxilla carcinoma, a finding further confirmed by the presence of a TP53 pathogenic mutation in T0. The same variant recurred four months later (T2) in association with clinical relapse (**Figure 3B**). In this scenario, the concentration of sDNA is even inversely proportional to the manifestation of disease, as we see the presence of a peak of sDNA within the only sample where no mutations were detected.

Lastly, HNSCC_05 is a 63 years old male diagnosed with squamocellular carcinoma in August 2022. After surgery, a significant drop in the amount of sDNA was assessed until T4, where a higher sDNA concentration was matched to the assessment of both a frameshift TP53 variant (p.Phe212SerfsTer3) and a missense FBXW7 mutation (p.Arg505Cys) (**Figure 3C**). Our approach was able to detect and increase in sDNA concentration and/or the presence of a stable deleterious SNV approximatively two months before conventional clinical evaluations. Although our data showed that in the vast majority of cases sDNA concentration drops dramatically following surgical resection of the primary lesion, we see that it tends to remain unchanged or at least lower even in the presence of disease flare-up (**Supplementary Figure S1**). Therefore, sDNA quantification, while providing important information for patient monitoring, must be supported by molecular characterization, since an increase in its concentration and the presence of a molecular recurrence do not always correspond. Indeed, our data support the idea that the solely amount of sDNA is often not sufficient to suspect the presence of a recurrence, which is instead assured evaluating a recurrent deleterious variant. Moreover, the workflow detailed in this study is able to detect somatic variants even in very low concentrations of sDNA.

Overall, our findings should be validated in longitudinal studies with larger sample sizes for a better understanding of the potential clinical impact of sDNA analysis as prognostic biomarker in monitoring the response to therapy in HNSCC.

Taken together, our data indicate that sDNA analysis could aid in the monitoring of patients’ follow-up as low-frequency somatic mutations could be assessed from the saliva of cancer patients. We are aware that our results are flawed by the reduced number of patients enrolled. Notwithstanding, these results suggest that salivary-based liquid biopsy could be capable of forerunning clinical relapse, shaping the management of those patients for whom surgery is the solely treatment and paving the way for personalized molecular therapies.

## Data Availability

The original contributions presented in the study are included in the article/**Supplementary Material**. Further inquiries can be directed to the corresponding author.
