# Supplementary material for: Longitudinal detection of somatic mutations in the saliva of head and neck squamous cell carcinoma–affected patients: a pilot study
Source: Front Oncol. 2024 Nov 1;14:1480302. doi: 10.3389/fonc.2024.1480302 (PMC11564150; doi:10.3389/fonc.2024.1480302)
Supplement: Supplementary file 1 [file DataSheet1.docx]

Supplementary Material

# Supplementary Figures and Tables

## Supplementary Figures

**
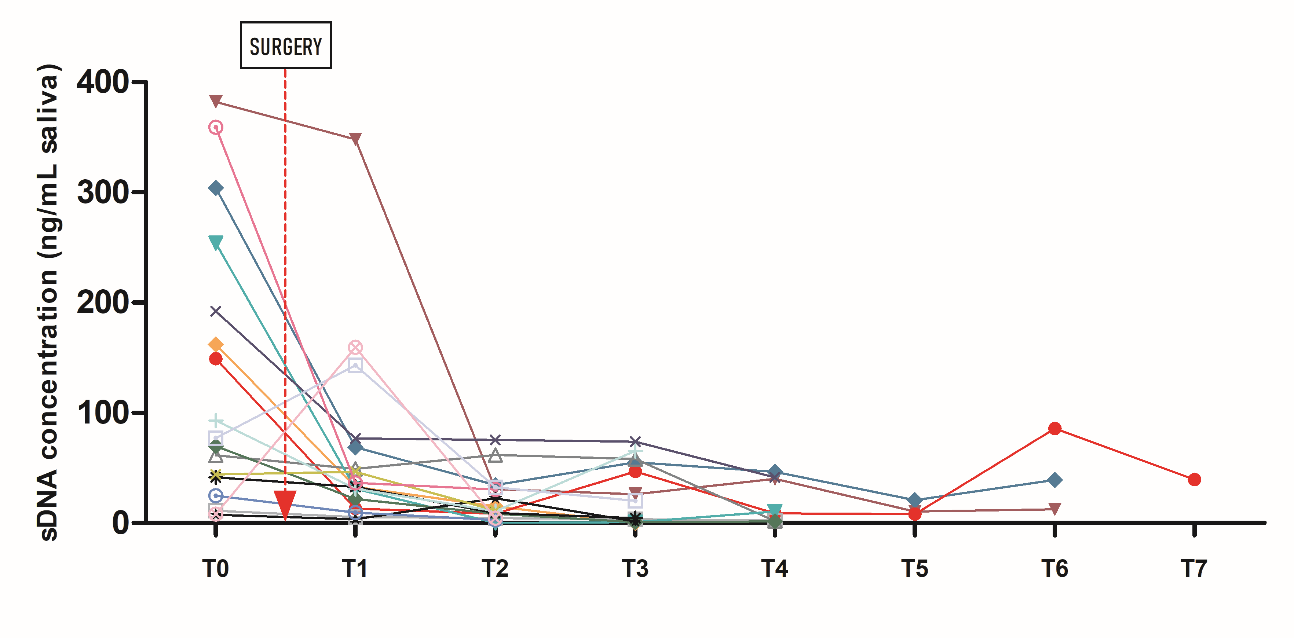
**

**Supplementary Figure 1:** **Outline of saliva sample collection.** For each patient enrolled in this study the first saliva sample was collected before surgery (T0) and it represents the sample with the highest concentration of salivary DNA due to the high disease burden. Subsequent saliva samples were collected during the subsequent visits according to the follow-up schedule (i.e., every 3 or 6 months).

## Supplementary Table

**Supplementary Table 1:** Risk factor details.

**Supplementary Table 2:** Detailed information about the alterations assessed in our cohort.
